# Unexpected retinal fluid compartment responses with anti-VEGF therapy in polypoidal choroidal vasculopathy: a case report of four patients

**DOI:** 10.3389/fopht.2024.1509065

**Published:** 2024-12-18

**Authors:** Noa Gilead, Yu Jeat Chong, Farah N. I. Ibrahim, Christopher Sun, Kelvin Y. C. Teo, Chui Ming Gemmy Cheung

**Affiliations:** ^1^ Singapore Eye Research Institute, Singapore National Eye Centre, Singapore, Singapore; ^2^ Medical Retina Department, Singapore National Eye Centre, Singapore, Singapore; ^3^ Ophthalmology and Visual Sciences Academic Clinical Program, Duke-NUS Medical School, Singapore, Singapore

**Keywords:** polypoidal choroidal vasculopathy (PCV), subretinal fluid (SRF), pigment epithelial detachment (PED), unexpected response, retinal fluid compartments, case report

## Abstract

This case series describes responses to faricimab treatment in opposite directions in different fluid compartments in four patients with polypoidal choroidal vasculopathy (PCV). Despite reductions in retinal fluid (SRF) and stable visual acuity following treatment, all patients developed retinal pigment epithelium (RPE) elevation. Over a 12–15 months follow-up, three patients exhibited a gradual decrease in RPE elevation, with one case resolving completely. These findings suggest that fluid compartments in PCV may respond differently to treatment and add to the understanding of PCV by highlighting the complex interplay between different retinal fluid compartments in response to treatment.

## Introduction

Polypoidal choroidal vasculopathy (PCV) is a subtype of neovascular age-related macular degeneration (nAMD) characterized by abnormal blood vessel growth beneath the retina, leading to polypoidal lesions and fluid accumulation. PCV often presents with subretinal fluid (SRF), intraretinal fluid (IRF), and pigment epithelial detachments (PED), contributing to vision loss in affected patients.

Faricimab is a relatively new treatment agent that has demonstrated effectiveness in treating nAMD, including PCV, by significantly improving visual acuity and retinal anatomy (1). Its bispecific mechanism of action targets both VEGF-A and angiopoietin 2 (Ang2), aiming to control neovascularization and enhance vessel stability (2). In the *post hoc* analysis of pooled data from the TENAYA and LUCERNE phase 3 trials comparing intravitreal faricimab to intravitreal aflibercept during the head-to-head dosing period, the faricimab-treated eyes showed greater reductions in all fluid compartments including subretinal fluid (SRF), intraretinal fluid (IRF) and serous pigment epithelial detachments (PED) (3). In a retrospective study of 63 eyes with wet AMD, including those with PEDs, faricimab loading therapy (three monthly injections) resulted in PED resolution in 24% of eyes and decreased PED height in 12% of eyes with baseline PEDs after 3 months (4).

Furthermore, a recent study by our group examining the response of switching to faricimab in nAMD patients found that eyes with serous PED or type 1 macular neovascularization (MNV) showed a significant reduction in maximum PED height following faricimab treatment. In contrast, eyes with non-serous PED or PCV exhibited a lower, non-significant reduction in mean PED height. These findings suggest that faricimab’s effect on PEDs may vary depending on specific disease characteristics (5). However, none of the previous studies had evaluated responses in the different fluid compartments in the same eye. Sub-retinal pigment epithelium (RPE) fluid is widely accepted as the most resistant compartment (6). Hence, there are often eyes that show improvement in SRF or IRF, but sub-RPE fluid persists. However, a response in the opposite direction in the three fluid compartments is not commonly expected and has rarely been evaluated.

In this case series, we describe four eyes with PCV treated with faricimab monotherapy, which experienced unexpected responses according to fluid compartment, with a reduction of fluid in one retinal compartment and a corresponding increase in fluid in another retinal compartment. Specifically, we observed improvement in SRF but an increase in PED. We further discuss the potential mechanisms that may explain this type of response.

## Case series summary

### Patient demographics and clinical presentation

We describe four patients with PCV diagnosis confirmed by multimodal imaging. There were two males and two females, aged between 65 and 73 years. None of the patients had a history of hypertension or diabetes mellitus. Faricimab was used as first-line therapy in one case. In the remaining three cases, two patients received prior treatment with aflibercept, and one was treated with ranibizumab (**Table 1**).

**Table 1:** Patient demographics and treatment history.

Case	Age	Gender	Diagnosis	Prior intravitreal injections	logMAR BCVA at first faricimab dose	Last interval pre switch (weeks)	Unexpected response timing	logMAR BCVA at detection of unexpected response	Subsequent treatment	Final visit logMAR BCVA
1	65	Female	PCV	Treatment naïve	0.3	Not Applicable	4 weeks post 1^st^ faricimab	0.2	6 faricimab	0
2	66	Male	PCV	3 ranibizumab	0.35	4	4 weeks post 1^st^ faricimab	0.2	4 faricimab 2 aflibercept	0.26
3	69	Female	PCV	>10 aflibercept	0.5	6	9 weeks post 3^rd^ faricimab	0.45	1 faricimab 7 aflibercept 5 faricimab	0.45
4	73	Male	PCV	12 aflibercept	0.25	6	5 weeks post 4^th^ faricimab	0.2	7 aflibercept 4 faricimab 4 aflibercept	0.3

**Figure 1:**
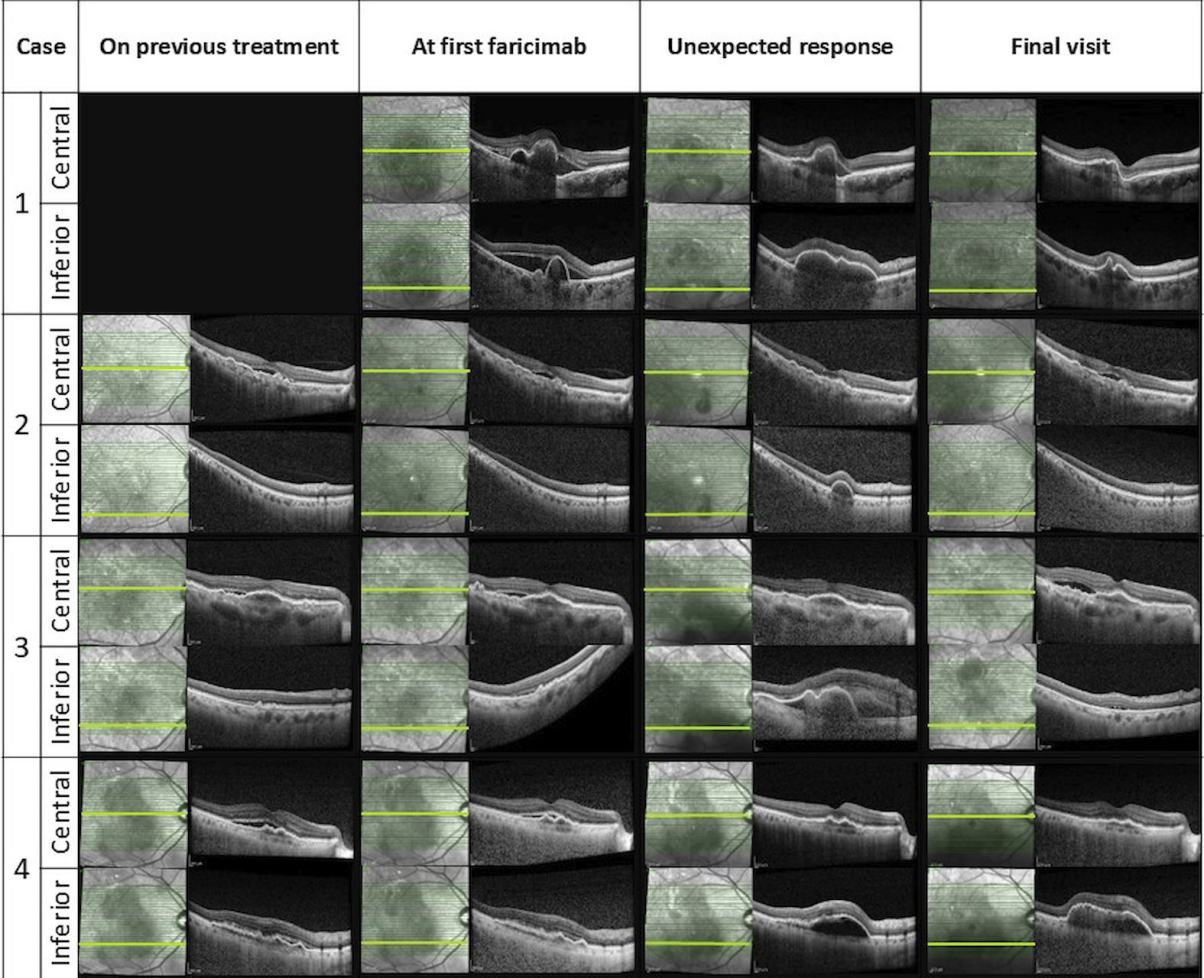
Changes in OCT central and inferior b scans over time.

After the first faricimab treatment, all eyes experienced a reduction in SRF and improved visual acuity at 4-6 weeks, with mean (SD) central macular thickness (CMT) decreasing from 453 µm (174) to 345 µm (150) and mean (SD) logMAR best corrected visual acuity (BCVA) improving from 0.4 (0.11) to 0.21 (0.18). However, inferior macular PED worsened either at the time of the first faricimab injection or during subsequent follow-ups. The worsening of inferior macular PEDs was noticed in all patients after 1-4 faricimab injections and 4-9 weeks from the last treatment given. One patient experienced subretinal and sub-RPE bleeding, and indocyanine green angiography (ICGA) showed the development of a new polypoidal lesion. Unexpectedly, the fovea center remained fluid-free in all cases despite the worsening of the extrafoveal PED. A summary of the change in OCT parameters and OCT imaging before and after this unexpected response are demonstrated in **Table 2** and **Figure 1** respectively.

**Table 2:** Summary of change in OCT parameters before and after unexpected response was detected.

Case	CMT (µm)				Max PED height (µm)				Max PED width (µm)			
	Previous treatment^1^	Pre switch	At unexpected response	Final visit	Previous treatment	Pre switch	At unexpected response	Final visit	Previous treatment	Pre switch	At unexpected response	Final visit
1	NA	708	566	280	NA	446	547	246	NA	1366	4607	2313
2	296	316	232	260	115	115	264	172	368	384	1211	1242
3	NA	385	414	358	NA	42	664	0	NA	302	3546	0
4	459	402	279	305	122	144	352	382	643	1051	3253	4468

1-Previous treatment includes data on the day of last anti-VEGF injection before the switch to faricimab.

NA, Not Applicable.

All patients were followed for a period of 12-15 months following the their unexpected responses to faricimab injection. Two patients continued on faricimab, while the other two were switched back to aflibercept, all under a treat-and-extend (T&E) protocol. At their final visit, the mean (SD) CMT was 301 µm (42.37), and the mean (SD) BCVA was 0.25 (0.18). The detailed course of disease of each patient has been summarized below (**Table 1**).

#### Case 1

A 65-year-old female presented with blurred vision in her right eye, with a logMAR BCVA of 0.30 and a CMT of 708 µm. Her ICGA at presentation revealed a cluster of polypoidal lesions subfoveally and another small polypoidal lesion inferiorly.

Unexpected response occurred 4 weeks after the first faricmab injection, when she developed significant enlargement of inferior macular PED despite a reduction of SRF and PED height in the fovea as observed on OCT (CMT decreasing from 708 to 566 µm).

**Figure 2:**
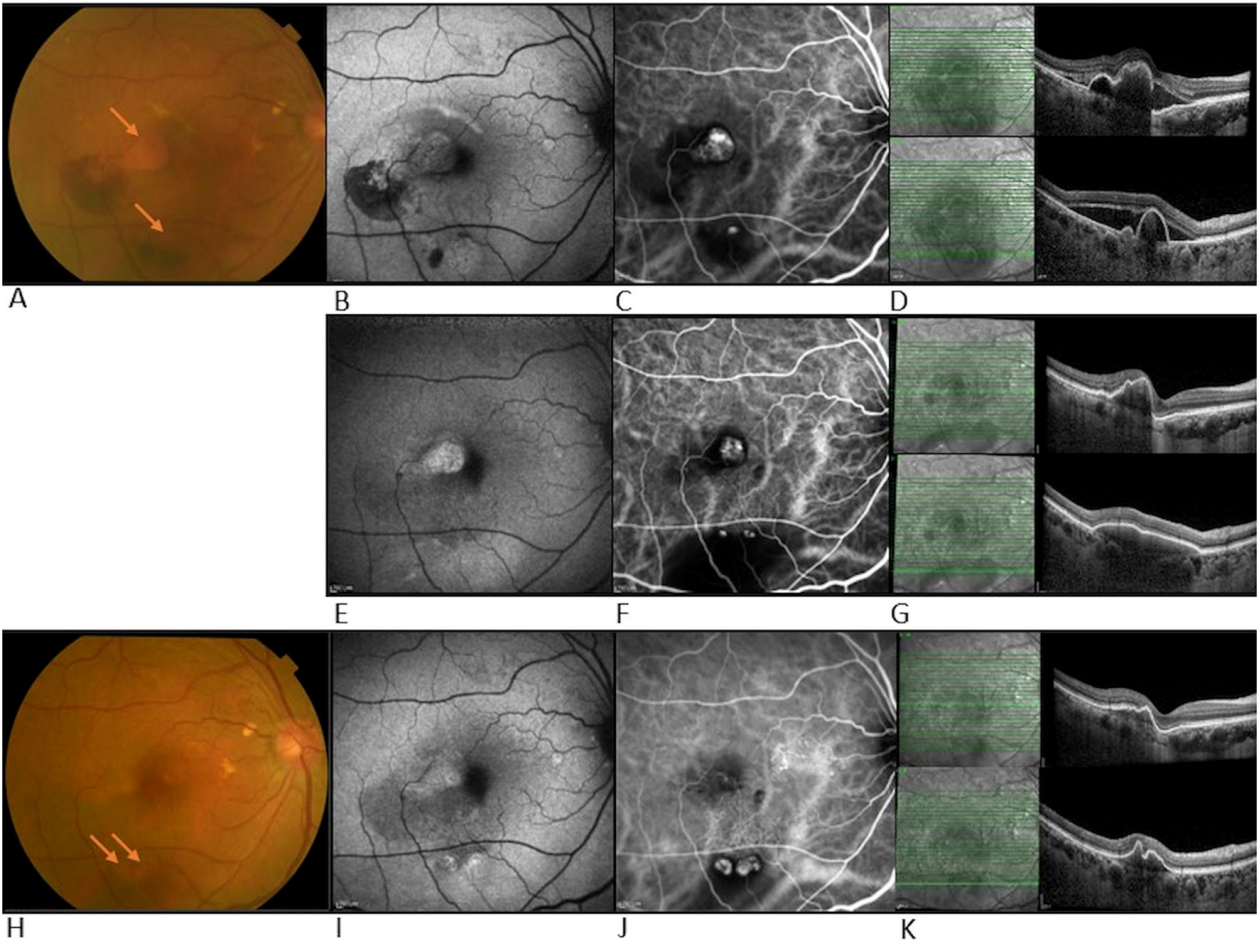
Multimodal imaging at diagnosis and after unexpected response in case 1. Top row- multimodal imaging at diagnosis of PCV: **(A)** Color fundus photograph (CFP) showing temporal and inferior bleeding and two adjacent orange polypoidal nodules (arrows), **(B)** Fundus autofluorescence (FAF), hypo-autofluorescence correlates with bleeding areas, **(C)** ICGA shows hyper-fluorescence suggesting leakage from both sites indicative of polypoidal lesions, **(D)** OCT shows SRF and PED in both central and inferior macula. Middle row- multimodal imaging after unexpected response: **(E)** FAF showing resolution of hemorrhage at central location, **(F)** ICG showing reduction in size of central polypoidal lesion but development of new polypoidal lesions inferiorly, and increased in surrounding dark area correlating with the large PED, **(G)** OCT b scan shows central SRF reduction and increase in size of inferior PED. Bottom row- multimodal imaging at week 52 follow-up: **(H)** CFP showing central hypopigmentation and two inferior orange nodules (arrows), **(I)** FAF showing slight hyper-autofluorescence temporal to fovea and inferior to macula, **(J)** ICGA shows regression of polypoidal lesions centrally but increase in the size of the two inferior polypoidal lesions, **(K)** OCT shows SRF has fully resolved and inferior PED shrunk.

She continued with faricimab treatment completing four initial loadings followed by a T&E regimen at 16 weeks intervals. Four weeks after her fourth faricimab, her CMT had reduced further to 411 µm, and SRF had completely resolved. Repeat ICGA at this point, however, showed new polypoidal lesions inferiorly despite a reduction in the area of the subfoveal polypoidal lesion. Over a follow-up period of 12 months, there was no recurrence of SRF, and her CMT gradually reduced to 280 µm. The height and width of the inferior PED decreased, and her BCVA was maintained at 0.00. Repeat ICGA at month 12 showed that the polypoidal lesions at the fovea center had regressed completely. However, two polypoidal lesions along the inferior arcade had increased further in size compared to week 16. Nonetheless, there were no SRF or IRF, and fluorescein angiography (FA) did not show leakage. Her latest treatment interval was 16 weeks. Multimodal imaging at diagnosis and after the unexpected response for this patient can be seen in **Figure 2**.

#### Case 2

A 66-year-old male with PCV and multiple polypoidal lesions nasal to the fovea, according to ICGA at presentation, received three monthly ranibizumab injections. He showed suboptimal response to ranibizumab and was switched to faricimab due to persistent SRF. At the time of switching, his BCVA was 0.35, and his CMT was 316 µm.

Unexpected response occurred four weeks after the first switch to faricimab, when an increase in inferior PED was observed despite the resolution of SRF (CMT decreased from 316 to 232 µm) and an improvement of visual acuity to 0.20.

He continued with faricimab treatment in 6-8 weeks intervals and, due to persistent fluctuating SRF, was switched again to aflibercept with good response. Over a follow-up period of 12 months, his CMT was maintained at 260 µm, although a sliver of residual SRF was tolerated. The height and width of the PED gradually decreased with ongoing faricimab treatment and further diminished after the switch to aflibercept. At the end of the follow-up, his visual acuity was recorded at 0.26.

#### Case 3

A 69-year-old female with a diagnosis of PCV in her right eye was previously treated with more than ten injections of aflibercept. Due to suboptimal response with persistent SRF, she was switched to faricimab. Prior to the switch, her logMAR BCVA was 0.50, and her CMT was 385 µm. Six weeks after her first faricimab injection, her SRF had completely resolved, her CMT decreased to 302 µm, and her BCVA improved to 0.3. She received the second and third doses of faricimab at 9-week intervals.

Unexpected response occurred nine weeks after her third faricimab injection. The patient reported increased distortion and a decrease in visual acuity to 0.45 was documented. Inferior macular sub-RPE bleeding and growth of inferior PED were noted despite a resolution of sub-foveal SRF. Repeat ICGA revealed an inferior polypoidal lesion with associated bleeding.

She was subsequently treated with a faricimab injection and focal laser therapy, resulting in a good response. Over a follow-up period of 13 months, she received seven aflibercept and four faricimab injections in a T&E regimen with intervals of up to six weeks, along with three subsequent focal lasers targeting the polypoidal lesion. Changes in treatment agents were guided by patient preferences, specifically influenced by financial considerations and concern over recurrence of the same phenomenon. At the end of the follow-up, her CMT was 358 µm with residual SRF, and the PED had fully resolved. Her visual acuity remained stable at 0.45 throughout the follow-up period.

#### Case 4

A 73-year-old male with a diagnosis of right eye PCV had been previously treated with 12 aflibercept injections at intervals of 4-6 weeks. Despite the short treatment interval he had persistent fluctuating SRF, with stable PED, and was subsequently switched to faricimab. Before switching, his logMAR BCVA was 0.25, and his CMT was 402 µm.

**Figure 3:**
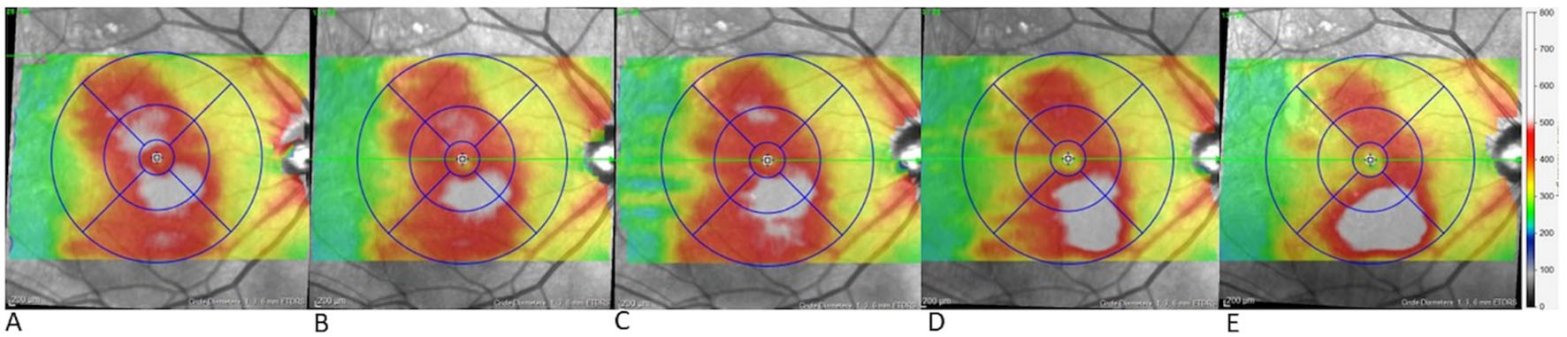
Gradual growth of inferior pigment epithelial detachment (PED) over time in case 4. Topographic map of the right eye in case 4 during the switch to faricimab **(A)** before the first faricimab injection, central and lower retinal thickening are present, **(B)** after the first faricimab injection, central thickening has resolved, indicating the resolution of central SRF, while minimal inferior thickening suggests initial growth in the inferior PED, **(C-E)** show progressive inferior thickening following the 2nd, 3rd and 4th faricimab injections respectively, with substantial growth observed after the 4th injection.

An unexpected response with increase of inferior PED occurred gradually, starting four weeks after the first faricimab injection and becoming significantly pronounced five weeks after the fourth faricimab injection, this is illustrated in **Figure 3**. The increase in inferior macular PED was observed despite resolution of central SRF (CMT decreased from 402 to 279 µm).

He was switched back to aflibercept due to the inferior PED growth, although no SRF was observed. Over a follow-up period of 15 months, his main treatment remained aflibercept injections, with four faricimab injections given briefly for financial reasons. However, SRF recurred with faricimab, prompting a return to aflibercept, which resolved the SRF. The inferior PED did not regress, and no further SRF recurrences were noted. At his latest follow-up, his CMT was 305 µm, but his PED height and width had increased under injection intervals of five weeks. Despite these changes, his visual acuity remained relatively stable, with a final logMAR BCVA of 0.3.

## Discussion

This case series highlights four eyes that exhibited improvement in SRF at the fovea but showed worsening of extrafoveal PED after treatment with faricimab, uniquely identifying an unexpected response in different retinal fluid compartments. This finding challenges the conventional assumption that all fluid compartments respond uniformly to therapy, even if at different paces and magnitudes, and highlights the importance to observe sub-RPE changes as this may signal early PCV recurrence.

While sub-RPE fluid, or PED, is generally recognized as a difficult-to-treat compartment, it typically does not worsen with anti-VEGF therapy. In fact, studies indicate that PED size often decreases with treatment. For instance, a large-scale study using AI to quantify OCT changes in over 2,000 eyes undergoing anti-VEGF treatment for neovascular AMD found an overall reduction in PED size (7). Additionally, the *post hoc* analysis of the phase 3 registration trials for faricimab showed that overall, at all groups, regardless of treatment, there was a reduction in PED size. These findings, however, were shown for large groups and did not highlight any intra-eye changes.

Specifically for faricimab, the aforementioned registrations trials showed a greater reduction in PED in the faricimab arm versus the aflibercept arm (3). Furthermore, our group’s recent study on patients switched to faricimab found that PEDs in PCV patients typically remained stable or showed partial resolution, without any observed growth. In that study, while SRF and IRF could fluctuate or worsen, PEDs either improved or remained unchanged (5). This experience aligns with the broader evidence that faricimab tends to reduce or stabilize PED size, even in complex cases of PCV.

In our observations, the worsening of PED was consistently observed in an extrafoveal location along the inferotemporal arcade in all cases. The significance of this location relative to the fovea in influencing the observed responses remains unclear. Interestingly, one patient developed a new polypoidal lesion at an extrafoveal location under faricimab treatment (case 3), while another experienced growth and worsening of an existing extrafoveal polypoidal lesion (case 1). Remarkably, in both patients, the central polypoidal lesions resolved simultaneously when the peripheral lesions worsened or developed.

We suggest two possibilities for these observations. First, the dual inhibition of Ang2 and VEGF by faricimab (8, 9) may differentially influence the permeability and inflammatory responses in various retinal compartments, potentially leading to improved SRF absorption but an exacerbation of sub-RPE fluid due to complex Ang2-mediated pathways that are not yet fully understood. However, it remains unknown whether similar unexpected responses occur with other agents and whether Ang2 significantly contributes to this effect.

Secondly, eyes with PCV subtype may respond differently to the dual inhibitory effects of faricimab. PCV lesions consist of the branching vascular network (BVN) and the polypoidal lesion. The histopathology of these components may differ in anatomical makeup and chronicity. We previously suggested that the BVN may consist of a hypermature network, while the polypoidal lesion may consist of a less matured coil of vessels with outpouchings (10). It is possible that anti VEGF-Ang2 was more effective on the less matured polypoidal lesion and not as effective on the hypermature BVN. This differential in effectiveness on the two components of PCV would explain the resolution of the original central polypoidal lesion, and over time, an occurrence of new polypoidal lesion at a new location, a consequence of poorer control of the BVN.

However, it is important to note that this observation might reflect one of two things – either part of variation in natural history of PCV or an unexpected response to anti VEGF treatment. Nonetheless, the persistence of disease activity under active anti-VEGF treatment, alongside simultaneous improvement in other areas, is intriguing, and further studies and investigations are needed to determine which is true.

Regarding patient management, our results suggest that continuing faricimab treatment led to favorable outcomes in at least two cases. The benefits of improved central fluid status and visual acuity may outweigh the observed increase in sub-RPE elevation, which tends to decrease over time, offering a reassuring prognosis.

## Conclusion

This case series highlights the need for a deeper exploration of the underlying mechanisms driving treatment responses and their molecular signaling pathways and emphasizes the importance of assessing the regions outside the central fovea, particularly in cases with PCV.

## Data Availability

The data analyzed in this study is subject to the following licenses/restrictions: Access to the dataset is restricted in compliance with ethical guidelines and data protection regulations. Sharing of the data requires appropriate permissions and is limited to authorized personnel for research purposes only, in accordance with institutional and legal requirements. Requests to access these datasets should be directed to noa.gilead.gilhar@seri.com.sg.
